# Aflatoxin Biosynthesis Is a Novel Source of Reactive Oxygen Species—A Potential Redox Signal to Initiate Resistance to Oxidative Stress?

**DOI:** 10.3390/toxins7051411

**Published:** 2015-04-28

**Authors:** Ludmila V. Roze, Maris Laivenieks, Sung-Yong Hong, Josephine Wee, Shu-Shyan Wong, Benjamin Vanos, Deena Awad, Kenneth C. Ehrlich, John E. Linz

**Affiliations:** 1 Department of Food Science and Human Nutrition, Michigan State University (MSU), East Lansing, MI 48824, USA; E-Mails: roze@msu.edu (L.V.R.); lunohong@yahoo.co.kr (S.-Y.H.); weejosep@msu.edu (J.W.); wongs94@yahoo.com (S.-S.W.); vanosben@msu.edu (B.V.); awaddeen@msu.edu (D.A.); 2 Department of Plant Biology, Michigan State University (MSU), East Lansing, MI 48824, USA; 3 Department of Microbiology and Molecular Genetics, Michigan State University (MSU), East Lansing, MI 48824, USA; E-Mail: laivenie@msu.edu; 4 Southern Regional Research Center, Agricultural Research Service, USDA, New Orleans, LA 70124, USA; E-Mail: ehrlich8@gmail.com; 5 Center for Integrative Toxicology, Michigan State University (MSU), East Lansing, MI 48824, USA

**Keywords:** *Aspergillus parasiticus*, oxidative stress, secondary metabolism, aflatoxin, endosomes, redox signaling

## Abstract

Aflatoxin biosynthesis in the filamentous fungus *Aspergillus parasiticus* involves a minimum of 21 enzymes, encoded by genes located in a 70 kb gene cluster. For aflatoxin biosynthesis to be completed, the required enzymes must be transported to specialized early and late endosomes called aflatoxisomes. Of particular significance, seven aflatoxin biosynthetic enzymes are P450/monooxygenases which catalyze reactions that can produce reactive oxygen species (ROS) as byproducts. Thus, oxidative reactions in the aflatoxin biosynthetic pathway could potentially be an additional source of intracellular ROS. The present work explores the hypothesis that the aflatoxin biosynthetic pathway generates ROS (designated as “secondary” ROS) in endosomes and that secondary ROS possess a signaling function. We used specific dyes that stain ROS in live cells and demonstrated that intracellular ROS levels correlate with the levels of aflatoxin synthesized. Moreover, feeding protoplasts with precursors of aflatoxin resulted in the increase in ROS generation. These data support the hypothesis. Our findings also suggest that secondary ROS may fulfill, at least in part, an important mechanistic role in increased tolerance to oxidative stress in germinating spores (seven-hour germlings) and in regulation of fungal development.

## 1. Introduction

In fungi redox signaling with the involvement of reactive oxygen species (ROS) has been implicated in a large number of processes including differentiation, asexual and sexual reproduction, sclerotia development, and fungal-plant interaction [1,2,3,4,5,6,7,8,9,10,11]. ROS, and in particular superoxide radical, have also been reported to control secondary metabolism [12]. Intracellular formation of ROS involves mainly mitochondrial oxidative phosphorylation, and reactions catalyzed by cell membrane, endosome NADPH oxidases (NOX), and enzymes for beta-oxidation of lipids in mitochondria and peroxisomes [3]. Oxidation reactions performed by monooxygenases/cytochrome P450 (CYP) isozymes, nitric oxide synthases, and xanthine oxidases also contribute to production of intracellular ROS [13,14,15,16,17,18,19,20]. Several studies provided evidence that CYP enzymes generate superoxide and hydrogen peroxide as intermediate compounds, and that these ROS can cause apoptosis and other cell pathologies [14,19,21,22,23].

Secondary metabolite pathways that are present in fungi and plants comprise a large number of CYPs and monooxygenases that carry out oxidative transformation of the intermediates in secondary metabolism. Consequently, these oxidative reactions could potentially be an additional uncharacterized source of intracellular ROS.

The filamentous fungus *Aspergillus parasiticus* is a focus of intense research effort due to its ability to synthesize the secondary metabolite and the potent carcinogen aflatoxin during growth on susceptible host plants. Aflatoxin biosynthesis occurs in endosomes/aflatoxisomes within the fungal cell; these compartments also enable storage and export of aflatoxin to the cell exterior [24]. The twenty-seven genes that encode aflatoxin biosynthetic enzymes are clustered; the entire aflatoxin biosynthetic pathway includes at least 21 enzymatic reactions [25]. Although aflatoxin biosynthesis is regulated by intracellular ROS of different origins [10,12], the aflatoxin biosynthetic pathway itself may serve as a source of ROS due to involvement of P450 enzymes. Indirect support for the idea of ROS production by aflatoxin biosynthetic pathway was provided by studies showing that an aflatoxin producing *A. parasiticus* strain generated significantly higher quantities of intracellular ROS than an aflatoxin non-producer [26]. Moreover, the same study demonstrated that genetic block in the aflatoxin biosynthetic pathway at an early, middle, or late step, correspondingly affected quantities of detected ROS.

In the current study we test the hypothesis that the aflatoxin biosynthetic pathway generates ROS which we designate “secondary ROS”. Since biosynthetic steps of aflatoxin biosynthesis are compartmentalized within endosomes/aflatoxisomes, these sub-cellular organelles could represent loci where secondary ROS is generated in association with distinct biosynthetic steps.

Using staining techniques which detect ROS in live *A. parasiticus* mutants that generate different quantities of aflatoxin, and feeding protoplasts with intermediates of aflatoxin biosynthetic pathway, we present data that support the hypothesis. The data suggest that the secondary ROS initiate redox signaling from endosomes/aflatoxisomes and that one downstream target of the signal is increased tolerance to oxidative stress in the next generation of germlings. This study contributes to our understanding of the role of secondary metabolism in fungal biology and helps to answer the question “why are secondary metabolites made?”

## 2. Results

### 2.1. Enzymes in Aflatoxin Biosynthesis Are a Potential Source of Secondary ROS

Table 1 lists the enzymes involved in aflatoxin biosynthesis that serve as potential sources of secondary ROS. At least seven enzymes encoded by *cypA* (*aflU*), *avnA* (*aflG*), *avfA* (*aflI*), *verA* (*aflN*), *ordA* (*aflQ*), *verB* (*aflL*), and *cypX* (*aflV*), belong to a superfamily of P450 monooxygenases that is capable of generating superoxide radicals. Among enzymes that participate in aflatoxin biosynthesis are two monooxygenases, aflX (*ordB*) and aflW(*moxY*). These enzymes are involved in early (*hypC* (38), *cypA*), middle (*verA*, *avnA*), and late steps (*ordA*, *cypA*, *moxY*, *ordA*) in the biosynthetic pathway.

**Table 1:** Enzymes and reactions that are potential sources of “secondary” reactive oxygen species (ROS) in aflatoxin biosynthetic pathway.

Gene ID in *A. flavus* genome database	Gene symbol (old gene symbol)	Gene/enzyme name	Reaction, reference
AFLA_139140	aflYa (nadA)	NADH oxidase	formation of AFG_1_ from HOMST [27]
AFLA_139160	aflX (ordB)	monooxygenase/oxidase	**-**
AFLA_139170	aflW (moxY)	monooxygenase	**-**
AFLA_139180	aflV (cypX)	cytochrome P450 monooxygenase	**-**
AFLA_139200	aflQ (ordA)	cytochrome P450 monooxygenase, CYP64	OMST to AFB_1_
AFLA_139230	aflI (avfA)	cytochrome P450 monooxygenase	oxidation of averufin to VHA
AFLA_139240	aflLa (HypB)	oxidase	oxidation of ST to AF (?) [28]
AFLA_139250	aflL (verB)	desaturase/P450 monooxygenase	**-**
AFLA_139260	aflG (avnA)	cytochrome P450 monooxygenase	AVN to HAVN
AFLA_139280	aflN (verA)	cytochrome P450 monooxygenase	VHA to ST
AFLA_139400	aflCa (HypC)	oxidase	NORA anthrone to NORA [28]
AFLA_139430	aflU (cypA)	cytochrome P450 monooxygenase	hydroxylate to NORA anthrone (?)

Abbreviations: AFG_1_, aflatoxin G_1_; HOMST, 11-hydroxy-*O*-methylsterigmatocystin; OMST, *O*-methylsterigmatocystin; AFB_1_, aflatoxin B_1_; VHA, versiconal hemiacetal acetate; ST, sterigmatocystin; AVN, averantin; HAVN, 5'-hydroxyaverantin; NORA, norsolorinic acid.

Moreover, *nadA*, a gene that encodes NADH oxidase, is located at the distal end of the aflatoxin cluster adjacent to the so-called sugar cluster. This gene encodes a protein that together with CypA catalyzes aflatoxin G formation [27,29]. The sugar cluster genes and *nadA* in particular, are co-regulated and transcribed in the same pattern as aflatoxin cluster genes [30,31,32]. NadA, like similar enzymes in bacteria, may contribute to generation of ROS and to alter the redox state [33]. Hence, a minimum of 10 enzymes associated with the aflatoxin biosynthetic pathway could potentially be a source of intracellular ROS.

### 2.2. Blocking Aflatoxin Biosynthesis Limits ROS Accumulation in Whole Live Mycelia of A. parasticus

**Figure 1:**
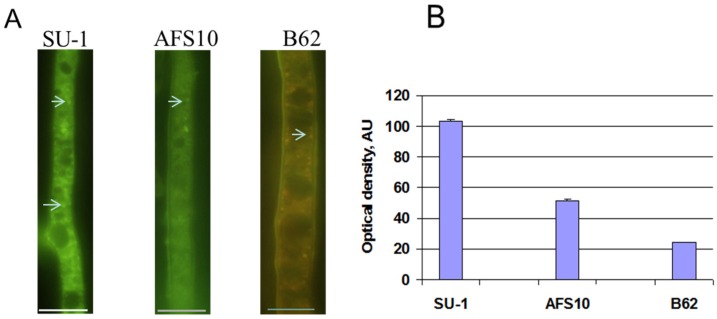

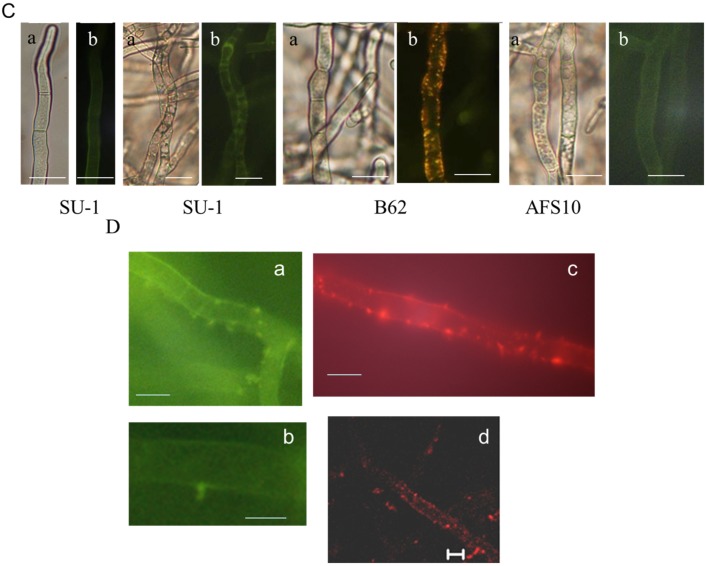
ROS detection using H_2_DCFDA in live *A. parasiticus* hyphae. *A. parasiticus* SU-1, AFS10, and B62 spores were inoculated at 10^4^ spores/mL into YES liquid medium and were grown at 30 °C, 150 rpm in the dark for 48 h. 6–8 colonies were incubated in the presence of H_2_DCFDA (20 µM) for 20 min, washed in PBS (0.02 mM, pH 7.5) for 5 min 4 times, and examined under fluorescent microscope. (**A**) Approximately 87% of SU-1, 43% of AFS10, and 48% of B62 hyphae contained structures of the size and shape of endosomes which stained green at different intensities with H_2_DCFDA (arrows; also see (**B**)). In strain B62 these structures accumulate NA, the early aflatoxin pathway intermediate; (**B**) Quantitative imaging analysis of ROS detected by H_2_DCFDA in *A. parasiticus* hyphae. AU, arbitrary units. Data are presented as the Mean ± SE, *N* = 8–12; (**C**) Autofluorescence of *A*. *parasiticus* SU-1, AFFS10, and B62 strains used in the study observed in the absence of staining by H_2_DCFDA: a, bright field image; b, image obtained under fluorescence microscope. Bar 100 µm; (**D**) Positive staining of protrusions with a fluorescent marker of autophagosomes and ROS. *A. parasiticus* SU-1 was grown for 66 h (as described in Figure 1) in the presence of Rapamycin (500 nM, added at 24 h to induce autophagy). 6–8 colonies were stained by Enzo Cyto-ID^®^ Green Detection Reagent according to manufacturer’s protocol. The stained cells were observed under a fluorescent microscope as described in Methods. The accumulation of the green fluorescent dye was detected in protrusions (**a**,**b**); *A. parasiticus* SU-1 grown for 48 h in liquid YES and stained with CellROX (**c**) or with FM4-64 (**d**). Bar 100 μm.

In attempt to detect ROS *in vivo*, ROS staining in live *A. parasiticus* hyphae was performed using H_2_DCFDA, a cell-permeable indicator for total ROS that is non-fluorescent until the acetate groups are removed by intracellular esterases and oxidation occurs within the cell. When *A. parasiticus* SU-1 (a wild type aflatoxin producing strain), is cultured in YES (yeast extract, sucrose) liquid medium in the dark for 48 h, it produces high levels of aflatoxin beginning at 30 h. AFS10 (carries a deletion in *aflR*, that encodes a key positive regulator of aflatoxin synthesis) produces no aflatoxin while B62 generates a bright red pathway intermediate norsolorinic acid (NA) and a small quantity of aflatoxin. At 48 h of growth, 38–95 individual hyphae were examined in each strain after staining with H_2_DCFDA. Approximately 87% of SU-1, 43% of AFS10, and 48% of B62 hyphae contained structures of the size and shape of endosomes (Figure 1A). These structures were stained green at different intensities with the highest intensity associated with endosomes in SU-1 and the weakest associated with endosomes in AFS10 (Figure 1A, arrows; Figure 1B). In B62 these endosome-like structures appear reddish-green due to accumulation of NA. In the corresponding controls (hyphae of SU-1, AFS10, and B62, with no H_2_DCFDA staining) we observed little detectable fluorescence under the microscope (Nikon, Inc., Melville, NY, USA) using B2A filter set (Figure 1C). We detected only weak autofluorescence in these strains and it was not associated with endosome-like structures. Moreover, B62 demonstrated bright reddish-yellowish small patches and protrusions were located predominantly along the cell wall and detected on the outer cell surface in agreement with our previous report that aflatoxisomes carry NA to the cell exterior (Figure 1A,C) [34]. Surprisingly, these patches and protrusions were also stained with a specific fluorescent probe for autophagosomes (Cyto-ID Autophagy Detection Kit, Enzo Life Sciences, Farmingdale, NY, USA) (Figure 1Da,b), with CellROX, another marker of total ROS (Figure 1Dc), and with a lipophilic styryl dye FM4-64 (Life Technologies, Molecular Probes, Eugene, OR, USA) that stains membranes (Figure 1Dd).

### 2.3. Disruption of AvaA (vb1, ypt7) Stimulates Aflatoxin Biosynthesis and ROS Accumulation in Whole Mycelia of A. parasiticus

**Figure 2:**
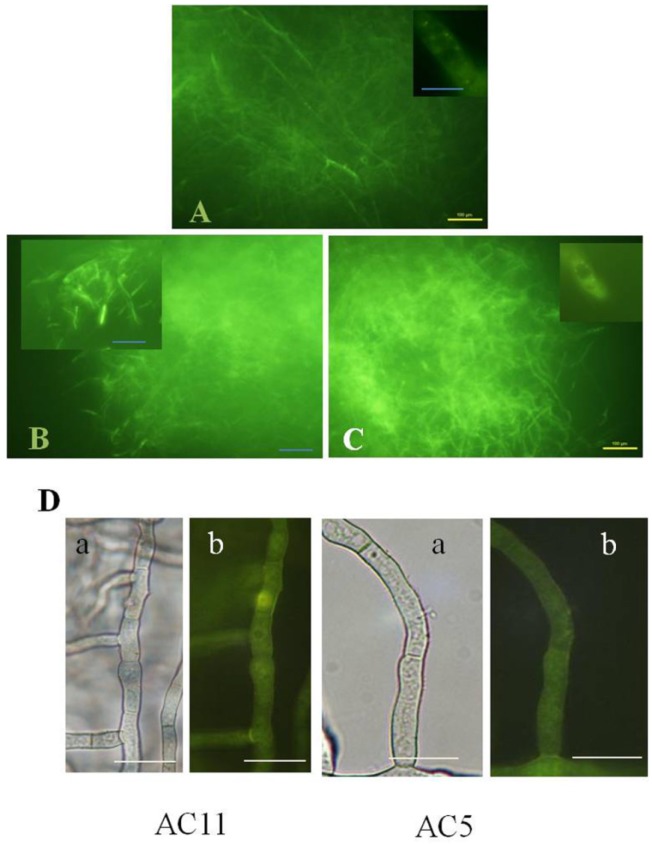
ROS detection using H_2_DCFDA in live *A. parasiticus* AC11 and AC5 hyphae. The strains were grown and stained as described in Figure 1. Small bright dots were detected in the cytoplasm and not in vacuoles. (**A**) *A. parasiticus* SU-1; (**B**) *A. parasiticus* AC11. Characteristic “scattered bright segment” pattern of staining in cells with bright cytoplasm; the overall brightness of staining in AC11 is higher than in SU-1; (**C**) *A. parasiticus* AC5. “Scattered bright segment” staining pattern; bright green small dots surround vacuole (Inset). The overall brightness of staining in AC11 and AC5 is higher than in SU-1. Bar, 100 µm; (**D**) Autofluorescence of *A. parasiticus* AC5 and AC11 strains used in the study observed in the absence of staining by H_2_DCFDA: (**a**), bright field image; (**b**), image obtained under fluorescence microscope. Bar 100 µm.

Disruption of *avaA* prevents fusion of pre-vacuolar compartments (late endosomes) to the vacuole and results in a significant increase in accumulation of endosomes and accumulation of aflatoxin in the growth medium [24]. AC11, a Δ*avaA* (Δ*vb1*) strain, was grown under the same as above conditions in aflatoxin-inducing medium (liquid YES). As expected, this strain produced larger quantities of aflatoxin B_1_ as detected by ELISA (2.7 mg/mL by 24 h of growth, and 9.3 mg/mL by 45 h) than SU-1 (2.49 μg/mL by 24 h, and 1.27 mg/mL by 45 h). At 48 h of growth, ROS accumulation in whole mycelia of *A. parasiticus* AC11 and AC5 (another Δ*avaA* mutant) using H_2_DCFDA, detected by fluorescence, was 2 to 3 fold higher than that in SU-1 (Figure 2A–C). AC11 and AC5 exhibited a characteristic “scattered bright segment” staining pattern of hyphae. Small brightly stained patches were detected in the cytoplasm and not in vacuoles in all of the strains examined. Such small brightly stained organelles exhibited the characteristic size and shape of transport vesicles and endosomes and they were found associated with the outer surface of vacuoles. AC11 and AC5 produced only weak auto-fluorescence in the absence of staining by H_2_DCFDA and this was not associated with endosomes (Figure 2D).

**Figure 3:**
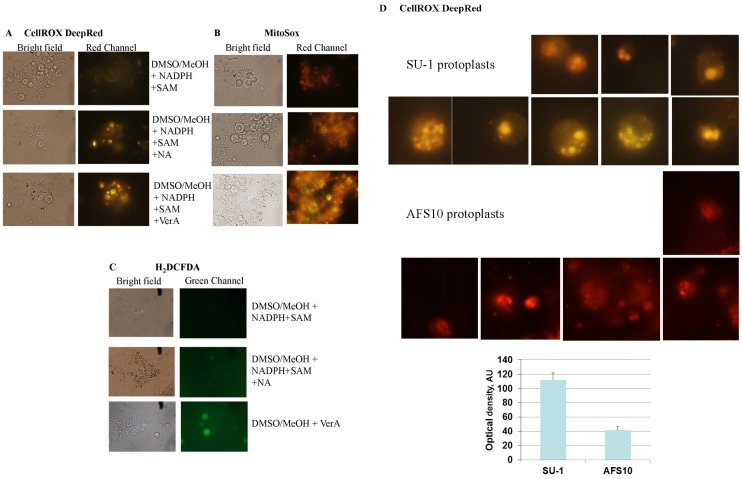
Feeding aflatoxin pathway intermediates to protoplasts stimulates intracellular ROS accumulation. Isolation and feeding of protoplasts were performed as described in Materials and methods. After feeding with NA and VerA the protoplasts were stained with CellROX Deep Red (**A**), Mitosox (**B**), and H_2_DCFDA (**C**), and visualized under a fluorescent microscope as described in Methods; (**D**) Comparison of the fluorescence intensity in SU-1 and AFS10 protoplasts stained with CellROX. SU-1 and AFS10 protoplasts were fed with VerA and stained using CellROX, as described in above. The images of individual protoplasts are presented in grayscale mode for assessment of the fluorescence intensity. For the graph the fluorescence intensity was assessed using ImageJ 1.48v software (NIH, Bethesda, MD, USA). AU, arbitrary units. Data are presented as the Mean ± SE, *N* = 6.

### 2.4. Feeding Aflatoxin Pathway Intermediates to Protoplasts Stimulates Intracellular “Secondary” ROS Accumulation

We obtained protoplasts from *A. parasiticus* SU-1 using standard methods [35] and fed them with the aflatoxin pathway intermediates NA (the first stable pathway intermediate) or versicolorin A (VerA, a middle pathway intermediate). Feeding with NA increased aflatoxin accumulation by 3-fold and feeding with VerA increased it by nearly 60-fold compared to control non-fed solvent added protoplasts. We analyzed intracellular ROS production in protoplasts using specific fluorescent dyes for superoxide (Mitosox) and for total ROS measurement (H_2_DCFDA or CellROX) after feeding for 16 h (Figure 3A–C). Although Mitosox is used to detect superoxide in mitochondria, our data indicate that Mitosox can also be oxidized to a fluorescent product by superoxide in endosomes. Feeding with pathway intermediates increased ROS accumulation to significantly higher levels than those of control protoplasts. The ROS signal detected with Mitosox, CellROX and H_2_DCFDA localized ROS accumulation to small organelles which exhibited the characteristic size and shape of transport vesicles and endosomes that were predominantly located near the cytoplasmic membrane. The increase in ROS was proportional to the increase in aflatoxin synthesis (*i.e.*, greater amounts of ROS were observed with VerA than with NA) (Figure 3A–C) suggesting that enzymes involved in aflatoxin synthesis, particularly CYPs, are directly or indirectly linked to increased ROS in transport vesicles and endosomes.

Formation of ROS after addition of NA or VerA may also derive from metabolic transformation of these compounds not related to aflatoxin biosynthesis. For example, in mammalian cell culture or tissue, aflatoxin B_1_ can be metabolized by CYPs or lipoxygenases with formation of free radicals [36,37]. In order to distinguish ROS generated through the aflatoxin biosynthetic pathway from ROS originated in non-aflatoxin-related metabolic transformation, we fed VerA to protoplasts obtained from *A. parasiticus* AFS10 in which no aflatoxin biosynthetic enzymes and no aflatoxin were present. AFS10 protoplasts fed with VerA were stained with CellROX, because it generates more easily detectable fluorescence as compared with H_2_DCFDA. Similar to SU-1 protoplasts, we detected ROS accumulation in AFS10 protoplasts in small organelles that exhibited a characteristic size and shape of transport vesicles and endosomes and to larger round structures; all these structures were located predominantly near the cytoplasmic membrane. However, the intensity of fluorescence (measured with ImageJ software, 1.48v) was 2–3-fold less in AFS10 protoplasts as compared to SU-1 (Figure 3D). The data suggest that aflatoxin biosynthesis is a primary contributor to ROS accumulation in endosome-like vesicular structures and that other source of ROS exist in these organelles as well.

### 2.5. Fungal Sensitivity to Hydrogen Peroxide Treatment Is Inversely Proportional to Aflatoxin Production

We analyzed and compared the levels of tolerance to hydrogen peroxide of conidiospores and germlings of different *A. parasiticus* strains that vary in the level of aflatoxin biosynthesis. Freshly harvested conidiospores and 7 h germlings of *A. parasiticus* SU-1, ASF10, AC11, HypC, and Δ*veA* were analyzed. These strains produce variable quantities of aflatoxin as expected depending on the mutation in the aflatoxin pathway. Of particular importance, ROS levels in these strains directly correlated with the quantities of aflatoxin produced (see above).

**Figure 4:**
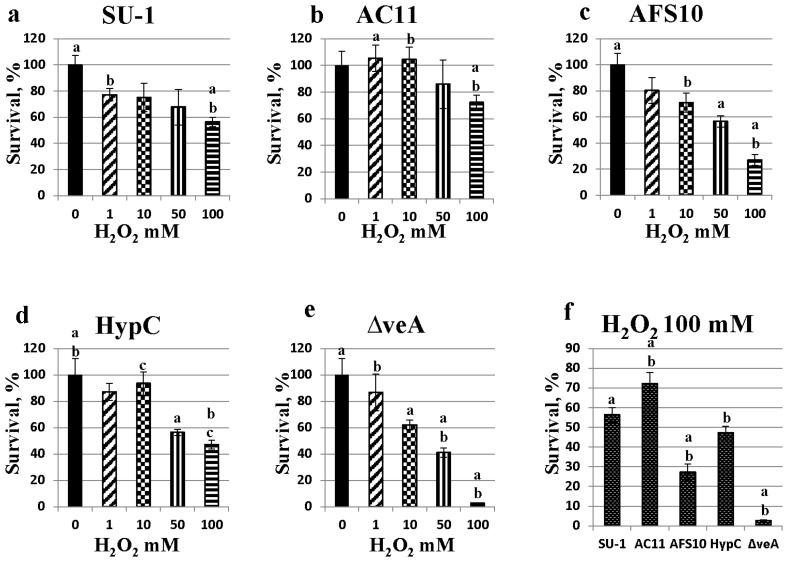
Tolerance of 7-h germlings to treatment with H_2_O_2_. *A. parasiticus* SU-1, AFS10, AC11, HypC, and *ΔveA* conidiospores (grown and freshly harvested as described in Methods) were incubated for 7 h in GMS liquid medium and then exposed to 1, 10, 50, or 100 mM hydrogen peroxide for 30 min. Viability of germlings was analyzed by growth of treated and untreated (control) germlings on YES agar growth medium which was incubated at 30 °C for 72 h. Fungal colonies were counted and % survival was calculated as follows: cfu treated/ cfu untreated × 100. Experiment was performed 5 times using 3 replicates with similar trends. Results of a representative experiment are presented as Mean±SE. An identical letter above the bar shows statistically significant difference in survival between the indicated data points (*p* < 0.05); statistical analysis was performed by pairwise Student’s t-test (SigmaStat) and by two-way ANOVA and Tukey’s HSD test (R Statistical Software, R-3.1.1).

We also observed clear differences in catalase activity in fresh spore suspensions of 3 *A. parasiticus* strains, SU-1, AFS10, and Δ*veA* (Table 2). In contrast to this observation, survival of freshly harvested spores from these 3 *A. parasiticus* strains, SU-1, AFS10, and Δ*veA*, under treatment with H_2_O_2_ was not significantly different (data not shown) suggesting that hydrogen peroxide may not enter freshly harvested spores. In support of this idea, when we germinated freshly harvested spores of five different *A. parasiticus* strains for 7 h in five independent experiments, we observed unique and reproducible trends in response to oxidative stress. AC11 which produces the largest quantities of aflatoxin had the highest tolerance to all concentrations of hydrogen peroxide tested and Δ*veA* in which no aflatoxin is produced was the most sensitive to hydrogen peroxide treatment (Figure 4a–e). Overall, we observed a strong inverse correlation between the quantity of aflatoxin produced by the strain and the sensitivity to hydrogen peroxide. The largest differences in sensitivity to hydrogen peroxide treatment were observed at the highest concentration of hydrogen peroxide tested, 100 mM (Figure 4f), and SU-1 (produced 2.49 μg/mL of aflatoxin B_1_ by 24 h, and 1.27 mg/mL of aflatoxin B_1_ by 45 h) survived at 2-fold or higher levels than AFS10 or Δ*veA*.

**Table 2:** Catalase activity in fresh conidiospores. Catalase activity was determined in a suspension of freshly harvested conidiospores by an assay provided in Worthington Enzyme Manual as described in Methods. ). The experiment was performed 3–4 times. The activity was expressed in micromoles H_2_O_2_ degraded per min per 10^4^ spores at 25 °C and pH 7.0, and presented as Mean ± SE. ND not detected. *, statistically significant difference as compared to SU-1, *p* = 0.0002. Statistical analysis was performed by Student’s *t*-test using SigmaStat 1.0).

SU-1	AFS10	Δ*veA*
3.0 ± 0.1	* 1.6 ± 0.1	ND

**Table 3:** Primer sequences used for transcript analysis.

Gene	Primer sequence ^a^	PCR product ^b^ (bp)	Intron ^c^
Citrate Synthase AFLA_007020	F-5' TGCAGTCCGTTGCCTTCAATG 3' R-5' TAGCGTAGGCCTTGGCGAAAG 3'	518 (827)	5
Cat Spore AFLA056170	F-5' AATGTACAGTCCAGCAAGG 3' R-5' CGGGTGAAGATAGACAAAG 3'	537 (648)	2
Cat Mycelia AFLA090690	F-5' ATGACACATTCCTGACCTC 3' R-5' TCTCATTGTCACCATGAG 3'	456 (583)	2
SOD Mn AFLA033420	F-5' CATTCTCCCTCCCACCTCTC 3 R-5'TCCAGATGCCCTCCACATAC 3'	549 (676)	2
SOD Fe AFLA027580	F-5' TGGGAGAGTTCCAGAGCAAG 3' R-5' TGTCGATGCCTTTCGGAG 3'	530 (796)	3
SOD Cu/Zn cytosol AFLA068080	F-5' GAAGCTGTTCTCCAGGAC 3' R-5' GACACCAGGTGGAAGTTAC 3'	434 (492)	1

**^a^** F represents forward primers and R represents reverse primers; **^b^** The size of PCR products obtained using genomic DNA (gDNA) as template is depicted in parenthesi; **^c^** The number of introns spanned by primers.

**Figure 5:**
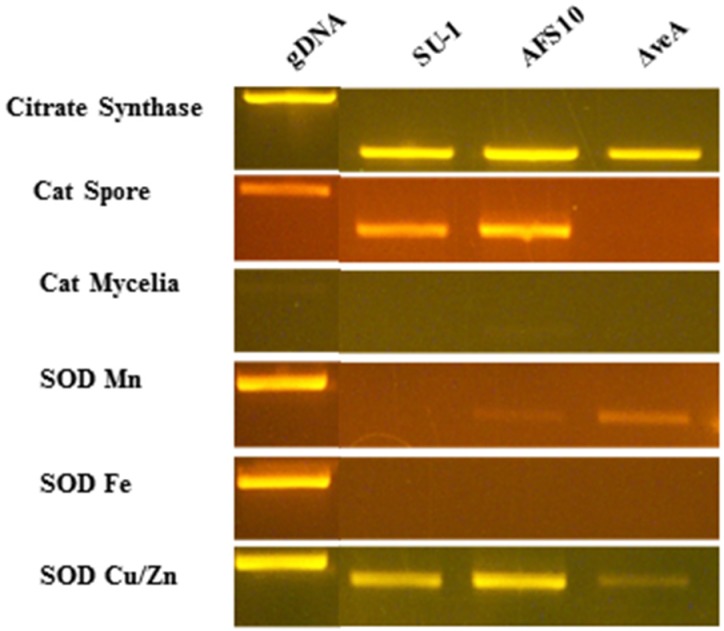
Analysis of transcript levels in 7 h germlings. *A. parasiticus* SU-1, AFS10, and Δ*veA* were grown on YES solid media and spores were collected at 5 days. Germlings were grown from fresh spores in GMS liquid medium for 7 h, frozen in liquid nitrogen, and stored at −80 °C. RNA was extracted from germlings using grinding and sonication as described in Methods. RT-PCR was performed on total RNA treated with RNAse-free DNAse I with primers specific for each gene (Table 3). PCR products were separated on a 1% agarose gel by electrophoresis. Citrate synthase, a constitutively expressed gene, was used as a positive control. gDNA, genomic DNA.

### 2.6. Transcript Accumulation in Seven-Hour Germlings

We measured total catalase activity in freshly harvested conidiospores in SU-1, AFS10, and Δ*veA*, and observed a direct correlation between catalase activity and resistance to hydrogen peroxide in 7 h germlings. However, we were unable to isolate RNA from conidiospores. To determine if this difference in catalase activity is regulated at the level of transcript accumulation, we analyzed expression of 3 catalases, catalase spore (AFLA_056170), catalase putative (AFLA_034380), catalase mycelial (AFLA_090690), and 3 superoxide dismutases, SODs, including SOD Mn (AFLA_033420), SOD Fe (AFLA_027580), and SOD Cu/Zn (AFLA_068080) in 7 h germlings of SU-1, AFS10, and Δ*veA* using semi-quantitative PCR. Catalase putative transcript was detected in *A. flavus* (68 h) but not in *A. parasiticus* strains studied (not shown). Disruption of *veA* caused a significant decrease in transcript accumulation of spore catalase and Cu/Zn SOD in 7 h germlings as compared to SU-1 and AFS10 (Figure 5). However, transcript accumulation of SOD Mn in Δ*veA* increased. Unfortunately, the expression data did not provide additional clues about the mechanisms of resistance to hydrogen peroxide in 7 h germlings.

## 3. Discussion

Increasing experimental evidence establishes that secondary metabolism contributes to the long term survival of the producing organism despite the fact that it can grow and reproduce in the absence of synthesis of secondary metabolites [38,39,40]. Aflatoxin biosynthesis requires a significant investment of energy, building materials, and the use of cellular organelles, in order to be completed. All these suggest that aflatoxins could be of biological importance to the producing fungus.

Our data provide support to the hypothesis that the aflatoxin biosynthetic pathway generates a redox signal in endosomes and this enhances resistance to oxidative stress in germinating conidiospores. We utilized mutants that produce different levels of aflatoxin, as well as specific dyes, Mitosox in particular, to provide data that suggest that the predominant ROS generated by the aflatoxin pathway is superoxide. Since superoxide radical is readily converted by SOD to hydrogen peroxide, the presence of hydrogen peroxide is also anticipated. Feeding experiments provided supporting evidence that the aflatoxin biosynthetic pathway generates ROS, presumably superoxide and hydrogen peroxide.

Secondary metabolism in *A. parasiticus* SU-1 generates multiple toxic and/or mutagenic compounds including aflatoxin, sterigmatocystin, and, as our data suggest, ROS. In order to protect itself from the damaging effects of these toxic chemicals, the fungus uses available cellular machinery such as compartmentalization, exocytosis, and antioxidant enzymes to manage exposure of the fungal cell to these compounds. Previous studies demonstrate that aflatoxin biosynthesis is carried out in specific sub-cellular compartments called aflatoxisomes and that these compartments also participate in storage and export of aflatoxin and other substances, such as aflatoxin enzymes, to the cell exterior [34]. The filamentous fungus *Fusarium graminearum*, a causative agent of head blight in wheat, apparently uses very similar compartments to fulfill biosynthesis of trichothecenes; these compartments have been named “toxisomes” and were shown to differ from peroxisomes [41]. Of particular importance, two enzymes involved in trichothecene biosynthesis, Tri4p and Tri1p, which are members of a P450 superfamily, co-localize in toxisomes. Future studies will determine if *Fusarium*, like *Aspergillus*, is capable of generating a ROS redox signal in toxisomes.

In the current study, vesicles that protrude through the cell surface outward were positively stained with CellROX and by a specific fluorescent marker of autophagosomes .These data suggest that autophagy may play a role in aflatoxin biosynthesis and autophagosomes may represent one sub-fraction of heterogeneous population of aflatoxisomes that participate in exocytosis of ROS and aflatoxins thus supporting the hypothesis. Antioxidant enzymes, catalase, and SOD, detected in endosomes/aflatoxisomes [42], may provide an additional control to the levels of ROS in endosomes.

Several previous studies suggested that aflatoxin biosynthesis participates in signaling functions. For example, aflatoxin biosynthesis intermediates were linked to sclerotia development; however, the mechanism of this regulation was not reported [25,43]. Similarly, conidiospore development and aflatoxin biosynthesis have been shown to be co-regulated [44]. Furthermore, biosynthesis of aflatoxin was not the only secondary metabolic pathway linked to development. Forseth and co-authors showed that two secondary metabolite clusters, *lna* and *lnb*, located on different chromosomes and containing two non-canonical non-ribosomal peptide synthetase (NRPS) genes, participate in regulation of sclerotia production in *Aspergillus flavus* [45]. Moreover, adducts of the secondary metabolites diorcinol and dehydroaustinol triggered initiation of sporulation in *A. nidulans* [46]. In addition, sclerotia development in *A. flavus* is responsive to ROS, in particular to hydrogen peroxide [10]. Our studies may provide a possible mechanistic link between the regulatory circuits for aflatoxin biosynthesis and development; this link is most likely secondary ROS generated by the biosynthetic pathway with the involvement of P450s.

It is clear that aflatoxin biosynthesis is regulated by oxidative stress (30–36). Recent studies by Grintzalis and co-workers demonstrated that a group of ROS including superoxide is involved in that regulation [10]. We speculate that secondary ROS may provide a feedback mechanism to self-activate the biosynthetic pathway.

Our data demonstrate a strong association between aflatoxin synthesis and resistance to oxidative stress (hydrogen peroxide) in germlings. The transcripts and the proteins encoded by aflatoxin biosynthetic genes are not detected in 7-h germlings but begin to accumulate in the fungal mycelium later, between 24 h and 30 h of growth [47]. The accumulation of transcripts and proteins correlates with accumulation of aflatoxin. For this reason, we suggest that pre-exposure of fungal cells to ROS generated in endosomes during aflatoxin biosynthesis in parental cells results in adaptation of cellular metabolism to promote increased tolerance to H_2_O_2_ in the next generation (young germlings). Therefore, our work may illustrate a novel function for aflatoxin biosynthesis. Specifically, the data suggest that aflatoxin biosynthesis provides protection and a competing advantage. In support of this idea, development of adaptive tolerance to stress by exposure to low levels of hydrogen peroxide was demonstrated previously in fibroblasts, bacteria, and plants [48,49,50,51].

**Figure 6:**
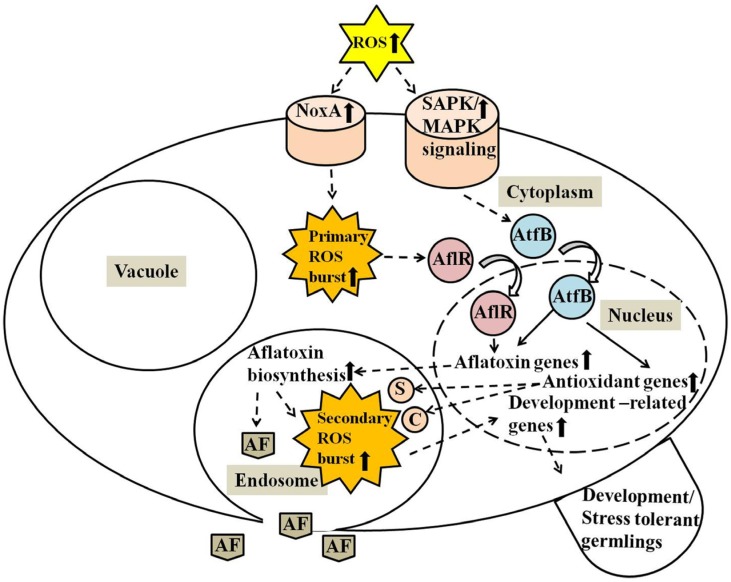
Secondary ROS and signaling from endosomes in aspergilli. Proposed model for stress-tolerant fungal germling development by ROS generated during secondary metabolism is based on available experimental data including current studies. The model proposes that increased levels of extracellular ROS up-regulate NADPH oxidase A (NoxA) and the stress activated protein kinase/mitogen activated protein kinase (SAPK/MAPK) signaling pathway. Activation of the SAPK/MAPK signaling cascade promotes AtfB binding to corresponding CRE sites in promoters of antioxidant and aflatoxin genes. NoxA induces intracellular primary ROS bursts that in turn activate AflR, which binds to corresponding AFLR sites in promoters of aflatoxin biosynthetic genes for activation of the gene expression. Aflatoxin biosynthetic enzymes are transported into endosomes, where aflatoxins (AF) are produced, and then secreted into medium concurrently with the toxin. Secondary ROS bursts produced during aflatoxin production in endosomes/aflatoxisomes, by most likely epigenetic mechanisms convey H_2_O_2_ resistance to next generation germlings. The dotted lined arrows indicate signal transduction and the solid lined arrows indicate binding of transcription factors, AtfB and AflR, to the corresponding sites in the promoters of the genes. The curved arrows designate entering of the transcription factors from cytoplasm to nucleus. Superoxide dismutase (S) and catalase (C) present in aflatoxisomes control levels of secondary ROS and associated signaling.

To summarize progress in our understanding of the role of ROS in fungi, we provide a regulatory model that emphasizes a key role for endosomes and associated secondary ROS in redox signaling (Figure 6). According to this model, extracellular ROS influence at least two signaling pathways. First, a NoxA initiated burst of ROS that we designate as “primary ROS burst”, modulates activity of AflR, a pathway-specific transcription factor that regulates aflatoxin biosynthesis. The SAPK/MAPK pathway, the second signaling pathway, activates the bZIP transcription factor AtfB, and promotes complex formation between AflR and AtfB at promoters of genes involved in aflatoxin synthesis, in promoters of antioxidant genes such as catalase and SOD, and promoters of genes involved in sclerotia/conidiospore development. Antioxidant and aflatoxin biosynthetic enzymes localize to transport vesicles which fuse to form endosomes. Activities of CYPs for aflatoxin biosynthesis, SOD, and catalase in endosomes generate and modulate a “secondary ROS burst” and subsequently a redox signaling from endosomes mediates development related processes (sclerotia and conidiospore development). The secondary ROS burst transmits increased tolerance to oxidative stress to the next generation of germlings. Endosomes eventually fuse with the cytoplasmic membrane to export their contents by exocytosis. Limitation for carbon stimulates aflatoxin synthesis and may also stimulate autophagy as aflatoxin synthesis is winding down when endosomes fuse with vacuoles.

Thus, secondary metabolism and secondary ROS in particular, may have the potential to regulate environmental fitness of the cell. Our work reveals the idea that aflatoxin biosynthesis-related secondary ROS may contribute to signaling in long term cell survival. Therefore, protection of cells from chemically unstable ROS (which makes the toxin) may have detrimental consequences to the survival of the cell.

## 4. Materials and Methods

### 4.1. Strains, Media, Growth Conditions

The *A. parasiticus* strains SU-1 (a wild type aflatoxin producing strain), *A. parasiticus* AFS10 (deletion in *aflR*, a key positive regulator of aflatoxin synthesis, produces no aflatoxin), *A. parasiticus* B62 (point mutation in *nor-1*, accumulates the bright red pathway intermediate norsolorinic acid (NA) and a small quantity of aflatoxin), *A. parasiticus* AC11 [24] (disruption of *avaA*, produces higher quantities of aflatoxin and increased toxin export of aflatoxin compared to SU-1), *A. parasiticus* Δ*hypC* [28] (accumulates smaller quantities of aflatoxin than wild type), and *A. parasiticus* Δ*veA* [52] (produces no secondary metabolites including aflatoxin) were used. YES, a rich medium, and GMS, a chemically defined medium, were used as aflatoxin-inducing media.

### 4.2. Detection of ROS in Live Cells

H_2_DCFDA, CellROX DeepRed and Mitosox were purchased from Invitrogen (Molecular Probes, Eugene, OR, USA). Whole mycelia and protoplasts were incubated with dyes in the growth medium for 10–15 min and washed in the medium 3 times according to the manufacturer’s instructions. Samples were observed and photographed under a Nikon fluorescence microscope using a filter with 495 nm excitation and 500–550 nm emission wavelengths (H_2_DCFDA and Mitosox staining) or a filter with 510 nm excitation and 580 nm emission wavelengths (CellROX Deep Red staining).

### 4.3. Measuring Catalase and SOD Activities

Activities of catalase and superoxide dismutase (SOD) were measured by protocols described in Worthington Enzyme Manual available online (http://www.worthington-biochem.com/CTL/assay.html). *Catalase activity assay*. The catalase assay is based on measuring the decrease of hydrogen peroxide levels during a reaction by means of a spectrophotometer (240 nm). The activity was expressed in μmol H_2_O_2_ degraded per min per milligram of total protein at 25 °C and pH 7.0.

To measure catalase activity using a crude protein extract from fungal mycelium, *A. parasiticus* spores were inoculated at 10^5^ spores/mL and grown in YES liquid medium for designated time in the dark with shaking at 150 rpm. The mycelium was harvested through Miracloth (Calbiochem, EMD Chemicals, San Diego, CA, USA) and frozen in liquid N_2_. Frozen mycelium was ground in liquid N_2_ in a mortar with a pestle and the powdered mycelium was resuspended 1:1 (*w*/*v*) in TSA buffer (0.01 M Tris, 0.15 M NaCl, 0.05% NaN_3_, pH 8.0) containing 1 tablet of Complete Mini Protease Inhibitor cocktail (Roche Diagnostics, Mannheim, Germany), 50 μL Proteinase Inhibitors mix (Sigma, St. Louis, MO, USA), and 0.125 mM phenylmethylsulphonyl fluoride (Sigma, St. Louis, MO, USA) per 10 mL. The cell lysate was centrifuged at 13,000 rpm at + 4 °C for 15 min. 50 μL of 1/10 diluted supernatant was added to a mix containing 0.95 mL of distilled sterile water and 0.5 mL of 0.059 M hydrogen peroxide in 0.05 M potassium phosphate (Mallinkrodt Baker, Phillipsburg, NJ, USA) pH 7.0. Decrease in absorbance at 240 nm was recorded using spectrophotometer Spectronic 601 (Milton Roy, Ivyland, PA, USA).

To assess catalase activity in conidiospores, freshly harvested spores (fungus was grown on GMS or YES agar medium at 30 °C, in dark (SU-1, AFS10), or light (Δ*veA*) for 7 days) were kept frozen at −80 °C before the assay as described above; 10^6^ or 10^7^ spores in 50 μL were used per reaction mix. The activity is expressed in micromoles H_2_O_2_/min/10^4^ spores.

*Superoxide dismutase activity assay*. The superoxide dismutase assay is based on measuring the inhibition rate of the production of blue formazan from nitro-blue tetrazolium reduction in the presence of superoxide. The maximum production of formazan was observed in the absence of the enzyme. The enzyme activity was expressed in Units per milligram of total protein, where one Unit is defined as enzyme quantity that is capable to inhibit nitro-blue tetrazolium reduction by 50%. Briefly, for each reaction mix, the following reagents were combined in a glass test tube: 0.1 mL of 0.1 M EDTA containing 0.3 mM NaCN (Mallinkrodt Baker, Phillipsburg, NJ, USA), 50 μL of 1.5 mM nitroblue tetrazolium (Sigma-Aldrich, St. Louis, MO, USA), 30 μL of cell lysate or spore suspension (or water in control), and 1.245 mL of 0.067 M potassium phosphate buffer, pH 7.8. The reaction was initiated by adding 75 μL of 0.12 mM riboflavin (Sigma-Aldrich, St. Louis, MO, USA) at timed intervals. The tubes were incubated on light for 12 min and the A_560_ was recorded every minute over 12 min interval.

### 4.4. Analysis of Aflatoxin Accumulation

Quantity of aflatoxin B_1_ in the growth medium was estimated using ELISA as described previously [30].

### 4.5. Analysis of Transcript Accumulation

At designated time points spores, germlings, or mycelia were frozen in liquid nitrogen and total RNA was extracted immediately from frozen cells using the Trizol reagent (Life Technologies, Carlsbad, GA, USA) and a protocol described previously [30]. RT-PCR was performed on total RNA treated with RNAse-free DNAse I (Roche Diagnostics, Indianapolis, IN, USA) with primers specific for the coding region of each gene. PCR products were separated on a 1% agarose gel by electrophoresis. The primer sequences used for transcript analyses are shown in Table 3.

### 4.6. Feeding Protoplasts with Aflatoxin Biosynthesis Intermediates

Protoplasts isolation and feeding with norsolorinic acid (NA) and versicolorin A (VerA) were performed as described previously [42]. Briefly, the protoplasts suspension (10^6^/mL) was aliquoted by 50 μL. The following compounds (as indicated) were added to the protoplasts: Control, 10 μL of DMSO/MeOH (1:1); 10 μL of 1 mM NADPH stock; 1 μL of 32 mM S-adenosylmethionine stock [53]; 40 µg of NA in 10 μL DMSO/MeOH; 243 µg of versicolorin A (VerA) in 10 μL DMSO/MeOH. NADPH and SAM are cofactors required for activity of enzymes in aflatoxin biosynthetic pathway [54,55]. The tubes with protoplasts were incubated at 30 °C for 1 h. All chemicals were from Sigma-Aldrich.

### 4.7. Assessment of Fungal Resistance to Hydrogen Peroxide Treatment

The first protocol describes treatment of conidiospores by hydrogen peroxide, while the second protocol was used to perform treatment of 7 h germlings. Protocol 1. *A. parasiticus* conidiospores (5 × 10^4^ spores in 50 µL) were spread onto YES agar medium and grown for 5 days at 30 °C; *A. parasiticus* SU-1 was grown in the dark while *A. parasiticus* Δ*veA* was grown in the light. Fresh spores (10^6^/mL) were harvested and treated in the presence of various concentrations of H_2_O_2_ for 30 min at 30 °C, washed two times with water, spread onto GMS agar plates, and the number of colonies was counted after 16 h of incubation at 30 °C in the dark. Protocol 2. *A. parasiticus* conidiospores (5 × 10^4^ spores in 50 µL) were spread onto YES agar medium and grown for 5 days at 30 °C; *A. parasiticus* SU-1 was grown in the dark while *A. parasiticus* Δ*veA* was grown in the light. Freshly harvested *A. parasiticus* conidiospores were inoculated into GMS liquid medium (10^4^/mL final concentration) and grown for 7 h at 30 °C with shaking at 150 rpm in the dark. Seven-hour germlings were incubated in the presence of various concentrations of H_2_O_2_ for 30 min at 30 °C in the dark, washed two times with water, spread onto GMS agar plates, and the number of colonies was counted after 16 h of incubation at 30 °C in the dark. *A. parasiticus* Δ*veA* was grown in the light.

### 4.8. Microscopy

For fluorescence microscopy, the samples were observed under a Nikon Labophot fluorescence microscope (Nikon Inc., Melville, NY, USA) using B2A filter set (EX 450–490 nm, EM above 520). The images were acquired with the Nikon Digital Sight Camera System (Nikon Inc., Melville, NY, USA). Quantitative measurements of the fluorescent signal of intracellular endosome-like structures were performed using grayscale mode and Adobe Photoshop 6.0 software (Adobe Systems Inc., San Jose, CA, USA). Mean values of 8–12 individual measurements were averaged and background (area containing no cells) was subtracted. Data are presented as Mean ± SE.

### 4.9. Statistical Analysis

Statistical analyses were performed by Student’s *t*-test using SigmaStat Scientific statistical software (version 1.0, Jandel Corporation, San Rafael, CA, USA) and by two-way ANOVA and Tukey’s HSD test (R Statistical Software, R-3.1.1, R Foundation for Statistical Computing, Vienna, Austria, 2013).

## 5. Conclusions and Perspectives for Future Studies

Our studies provide two main conclusions. (1) The aflatoxin biosynthetic pathway in *A. parasiticus* directly or indirectly generates ROS in aflatoxisomes/endosomes; and (2) Superoxide represents a significant fraction of the aflatoxin biosynthesis-derived ROS pool.

The ability of the aflatoxin biosynthetic pathway to generate ROS in endosomes with signaling function opens up a new direction for work on the secondary metabolism and its significance for development and cell survival. Although several fungal species are able to synthesize aflatoxins and structurally related compounds [47], fungal genomes include a large number of different secondary metabolic pathways in addition to the aflatoxin cluster [44,56]. Moreover, biosynthesis of secondary metabolites comprises various oxidative reactions with the participation of cytochrome P450s [57]. Most likely, the aflatoxin biosynthetic pathway is unique in the sense that it carries the highest number of cytochrome P450s among secondary metabolic pathways. Out of a total of approximately 150 ORFs identified as cytochrome P450s in the *A. flavus* genome, seven of these genes are present in the aflatoxin gene cluster (http://www.aspergillusflavus.org/genomics/). Hence, secondary metabolic pathways may provide continuous input to the intracellular pool of ROS and associated redox signaling. Future studies will attempt to uncover the specific mechanisms underlying secondary ROS signaling and its significance.
